# IL-6 induction in desiccated corneal epithelium in vitro and in vivo

**Published:** 2011-09-13

**Authors:** Akihiro Higuchi, Tetsuya Kawakita, Kazuo Tsubota

**Affiliations:** 1 Center for Integrated Medical Research, Keio University School of Medicine, Tokyo, Japan; 2 Department of Ophthalmology, Keio University School of Medicine, Tokyo, Japan

## Abstract

**Purpose:**

To investigate the effect of desiccation on secretion of inflammatory cytokines in corneal epithelial cells and in the rat desiccation model.

**Methods:**

A human corneal epithelial cell line (CEPI) was grown in keratinocyte growth medium 2 (KGM2) to approximately 80% confluence. The medium was aspirated and dishes were left for 0 to 30 min with the cover left open to dry the cells (short-term desiccation). After desiccation, KGM2 was added to the dishes and collected from the dishes 15 min later to measure the concentrations of cytokines in the medium by sandwich enzyme immunoassay (ELISA). Viability of the cells was estimated with alamer blue. To study the effect of long-term desiccation, cultivated cells on transwells were used. After dessiccation for up to 8 h, the viability of the cells and levels of cytokines in the culture medium were examined. The expression of cytokines in the cornea of the dry eye model rat was measured by real-time PCR.

**Results:**

Short-term dessication of CEPI cells significantly increased the interleukin (IL)-6 level and slightly increased the tumor necrosis factor (TNF)-α level. Anti-IL-6 antibody partially suppressed cell death caused by desiccation. Upon long-term desiccation, IL-6 and IL-8 levels were increased.  In the dry eye model rats, the *IL-6* mRNA level in the cornea significantly increased, whereas *TNF-α* mRNA level slightly increased.

**Conclusions:**

Desiccation induced IL-6 expression in corneal epithelial cells, suggesting that IL-6 participates in desiccation-induced cell death.

## Introduction

Cells live in an aqueous environment. Some mucosal epithelial cells, such as corneal epithelial cells, are directly exposed to the outside environment, and these cells are covered by an aqueous solution to keep the cells in a moist environment. Corneal epithelial cells are affected by various physical factors such as temperature [1,2], humidity [3,4], ultraviolet irradiation [5-7], and airflow [8]. Desiccation significantly affects these cellular conditions.

Tears are composed of three layers, namely, the mucin layer, liquid layer, and lipid layer, in order of the distance from the ocular surface. In severe dry eye, such as Sjogren’s syndrome (SS), the ocular surface becomes dry because tear secretion from the lacrimal glands decreases. This type of dry eye results not only from dryness of the ocular surface, but also from the lack of tear components, which are essential for maintaining the ocular surface [9-14]. The lipid layer defends against excessive evaporation of tears [15]. In some dry eye patients, tears evaporate excessively because of unusual components of lipid in the lipid layer, and the ocular surface becomes dry [16-18]. Corneal and conjunctival epithelial cells produce a group of highly glycosylated glycoprotein**s** termed mucin**s** [19]. Mucins provide a barrier against pathogen invasion, and due to their hydrophilic nature, prevent desiccation of the ocular surface [20]. Alteration of mucin secretion may cause dry eye [21,22].

There are many kinds of cytokines in tears, such as interleukin (IL)-1α, IL-1β, IL-6 [23], and tumor necrosis factor-α **(**TNF-α) [24]. Further more, recent study shows that twenty-five cytokines and chemokines were detected in tears from healthy subjects [25]. Cytokines may play physiologic roles in maintaining the ocular surface. Alteration on cytokines level in tears may be reflected by extraordinary in the ocular surface. In previous studies, significantly increased levels of *IL-1α*, *IL-6*, and *TNF-α* RNA transcripts were found in the conjunctival epithelium in SS and non-SS dry eye patients compared with controls [26,27]. The secretion of cytokines from the ocular surface in severe dry eye has been studied, but it is still unclear which cytokines are induced by excessive evaporation of tears. Evaporation of tears has been recognized as one of the key factors in dry eye diseases [28,29]. In this study, we report the effect of desiccation on inflammatory cytokine production in corneal cells using a human corneal epithelial cell line (CEPI), CEPI-17-CL4, and dry eye model rats.

## Methods

### Cells and culture

CEPI cells (CEPI-17-CL4 cells) were kindly provided by Dr. Kuwahara (Alcon Laboratories, Fort Worth, TX). These cells were immortalized cells infected with a recombinant SV40-retrovirus vector containing the Bg1I-HpaI fragment of SV40 T-antigen [30], and express an extensive array of cytokines, growth factors, and metabolic enzymes that resemble the original tissue [31]. CEPI cells were cultured in keratinocyte growth medium 2 (KGM2). The KGM2 was prepared by adding 30 μg/ml bovine pituitary extract, 0.5 μg/ml hydrocortisone, 5 μg/ml insulin, 0.1 ng/ml human epidermal growth factor, 10 μg/ml transferrin, 0.5 μg/ml epinephrine, 100 U/ml penicillin, 100 μg/ml streptomycin and 0.3 mM calcium chloride to Keratinocyte basal medium 2 (KBM2). KBM2 and the reagents that were added were purchased from Clonetic Corp. (San Diego, CA).

### Short-term desiccation

2×10^6^ CEPI cells were seeded in 0.1% gelatin-coated ϕ6cm dishes (Asahi Techno Glass Corporation, Tokyo, Japan) and cultivated overnight to attach the cells to the dish. The medium was discarded by aspiration and the dishes were left for 0, 5, 10, 15, 20, 25, and 30 min with the cover left open in the clean bench to desiccate the cells. KGM2 containing 10% alamarBlue (Trek Diagnostic Systems, Inc., Westlake, OH) was added to each dish to estimate the viability of the desiccated cells. Five or 50 ng/ml of anti-human IL6 antibody (BD Bioscience, San Jose, CA) was added to the medium and incubated for 1 h before desiccation to estimate the effect of anti-IL6 antibody on the survivability of CEPI cells during desiccation. After desiccation for the indicated period of time, KGM2 was added to the dishes to recover the cytokines secreted by the CEPI cells. Fifteen min later, the medium was collected and the concentrations of cytokines in the medium were measured by sandwich enzyme immunoassay **(**EIA). Captured and biotinylated antibodies, and recombinant human cytokines were purchased from BD Bioscience. Since the ELISA system prepared by us is highly sensitive, the limit of detection of the ELISA is approximately 0.01 ng/ml.

### Long-term desiccation

3×10^5^ CEPI cells were seeded on the membrane of ϕ2.4cm transwell with 0.4 μm pore size (Corning Inc., Corning, NY) and cultivated overnight to attach the cells to the membrane. The media in the under-layer and upper-layer were changed to 0.8 ml of fresh KGM2, respectively. To desiccate the CEPI cells, the medium in the upper-layer was discarded and the cells were incubated for 0, 2, 4, 6, or 8 h. After incubation for the indicated periods of time, medium was added to the upper-layer of each membrane in the transwell to recover the cytokines. The culture media in the upper-layer and lower-layer were collected and mixed to measure the concentration**s** of cytokines. One ml of KGM2 containing 10% alamarBlue was added to each of the upper-layer and lower-layer of each transwell to estimate the viability of the cells. The concentrations of cytokines in the culture medium were measured by the same methods as those described in the short term desiccation experiment.

### mRNA expression in CEPI cells upon long-term desiccation

After long-term desiccation using transwells, CEPI cells were harvested from the membrane of the transwell, and total RNA were extracted using GeneElute (Sigma-Aldrich Co., St. Louis, MO) to assay the mRNA expression of cytokines. Reverse transcription (RT) was performed using ReverTra Dash (TOYOBO CO., LTD., Osaka, Japan) according to the manufacturer’s protocol. PCR of the cDNAs of cytokines and glyceraldehyde 3-phosphate dehydrogenase (*GAPDH*) was performed using the Advantage 2 PCR kit (BD Bioscience). The primer sets for cytokines and *GAPDH* were purchased from TOYOBO CO., LTD.

TaqMan real-time RT–PCR of IL-6 and IL-8 was performed using the ABI PRISM 7700 Sequence Detection Systems (Life Technologies Corporation, Carlsbad, CA) according to the manufacturer’s protocol. Primer sets and TaqMan probes, and other reagents for TaqMan real-time PCR were purchased from Life Technologies Corporation. All data were analyzed with the ΔΔCt method and the mRNA of *GAPDH* was used as the internal standard.

### Animal model

All animal experiments were approved by the Laboratory Animal Care and Use Committee of Keio University School of Medicine. Four-week-old, male Sprague-Dawley rats (n=4 in each experiment) were purchased from Tokyo Laboratory Animal Science Co., Ltd (Tokyo, Japan). The rats were anesthetized by intramuscular injection of an anesthesia cocktail containing ketamine and xylazin. After deep anesthesia was induced, the eyelids of the rats were kept open by an adhesive agent. The rats were placed in a desiccation room that had a room temperature of 25±2 °C, relative humidity 50±2%, and constant airflow (1 m/s), and were maintained for 4 or 8 h. The ocular surfaces of the rats were photographed after applying a fluorescein solution under cobalt blue light. Since the amount of corneal epithelium is too small, we collect RNA from whole corneas. The corneas were collected from the rats after 0, 4, or 8 h of desiccation, and RNA was extracted from the cornea to measure the expression of IL-6 and TNF-α by real-time RT–PCR using ABI PRISM 7000 Sequence Detection Systems (Life Technologies Corporation) according to the manufacturer’s protocol. All reagents were purchased from Life Technologies Corporation and all data were analyzed same as CEPI cells on long-term desiccation in the method section.

## Results

### Cytokine induction in CEPI cells by short-term desiccation

**Figure 1:**
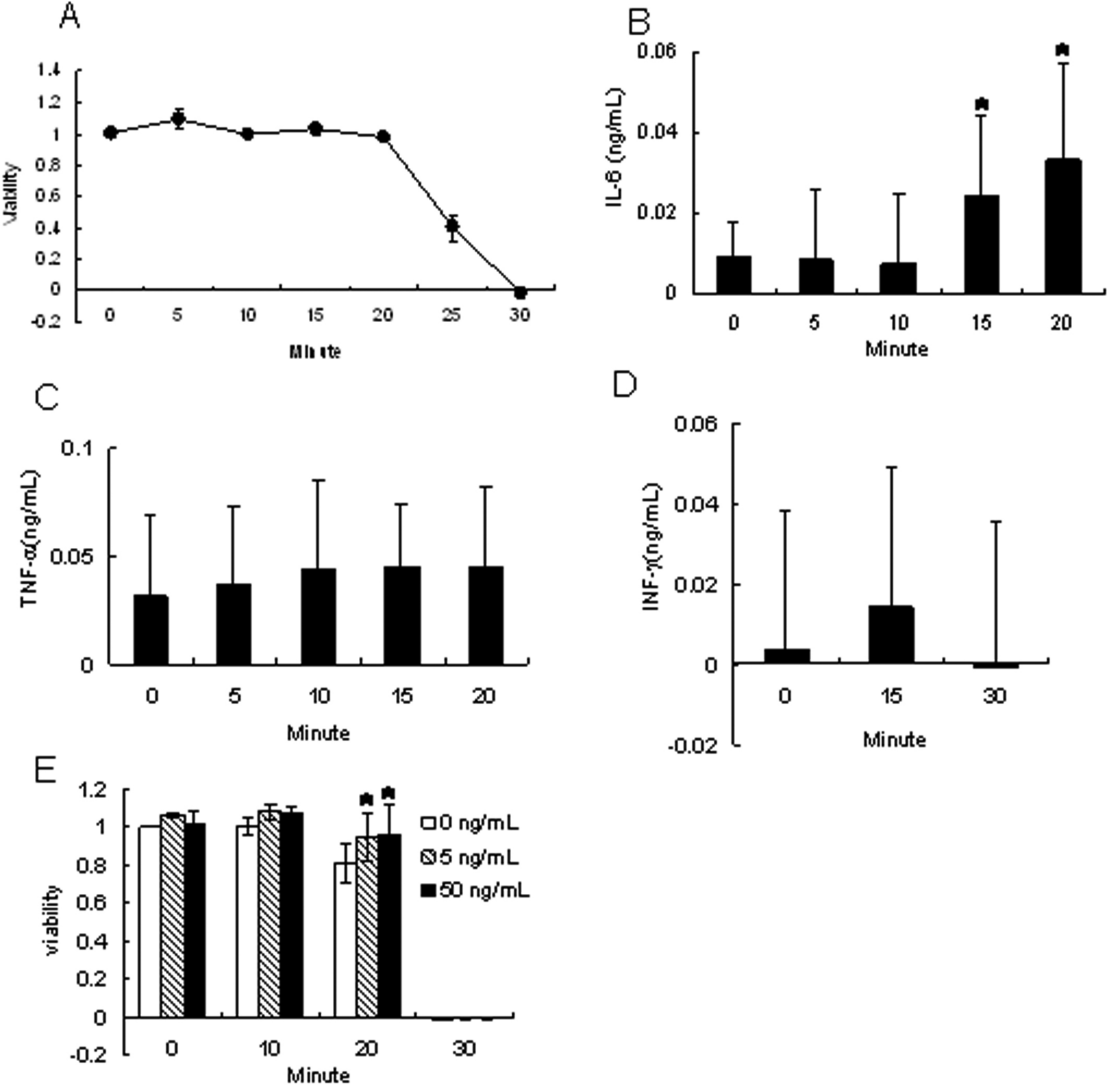
Effect of short-term desiccation on CEPI cells. CEPI cells were subjected to desiccation for up to 30 min. The horizontal axis shows the length of time that the cells were subjected to desiccation. **A**: Cellular viability of CEPI cells. **B**-**D**: Concentration**s** of IL-6 (**B**), TNF-α (**C**), or IFN-γ (**D**) in the medium secreted from CEPI cells. **E**: CEPI cells were incubated with 5 or 50 ng/ml anti-human IL-6 antibody for 1 h before desiccation to estimate the effect of anti-IL-6 antibody on the survivability of CEPI cells. Results are expressed as the mean±SD (n=6). Dunnett’s test was used to determine the significance of differences. The asterisk indicates a significant difference from the result at 0 min, p<0.05.

In the viability assay using alamarBlue, cell death was observed in CEPI cells that had undergone desiccation for 20–25 min (Figure 1A). The levels of cytokines in the culture medium were measured after desiccation for 0–20 min. The concentration of IL-6 in the medium after desiccation of CEPI cells for 15 or 20 min was significantly higher than that at baseline (desiccation time=0 min; p<0.05, p<0.05; Figure 1B). The concentration of TNF-α increased weakly after desiccation for 20 min (Figure 1C). IFN-γ expression was not induced by desiccation (Figure 1D). Treatment of CEPI cells with 20 ng/ml TPA and 0.1 μg/ml ionomycin for 72 h induced the secretion of IFN-γ (data not shown), indicating that CEPI cells maintained the ability to produce IFN-γ. Anti-IL6 antibody partially protected CEPI cells from cell death caused by desiccation (Figure 1E).

### Cytokine induction in CEPI cells by long-term desiccation

**Figure 2:**
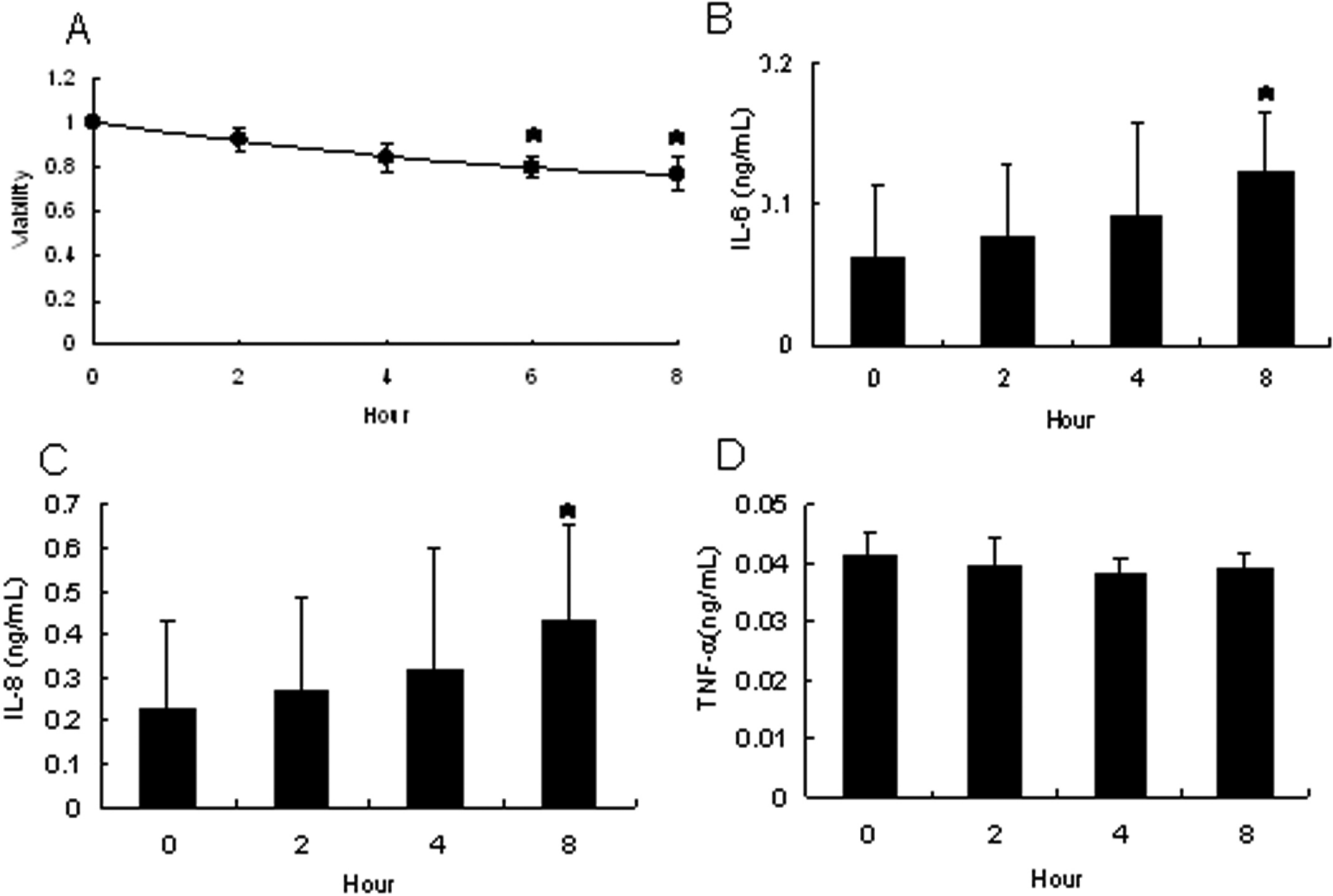
Effect of long-term desiccation on CEPI cells. CEPI cells were subjected to desiccation for up to 8 h. The horizontal axis shows the length of time that the cells were subjected to desiccation. **A**: Cellular viability of CEPI cells. **B**-**D**: Concentrations of IL-6 (**B**), IL-8 (**C**), or TNF-α (**D**) in the medium secreted from CEPI cells. Results are expressed as the mean±SD (n=6). Dunnett’s test was used to determine the significance of differences. The asterisk indicates a significant difference from the result at 0 min, p<0.05.

Since the influence of acute desiccation is not same as that of long-term desiccation, we study whether long-term desiccation induces the production of the same cytokines. CEPI cells were cultivated on the membrane and then desiccated for several h. Figure 2A shows the effect of desiccation on the viability of CEPI cells. Viability decreased upon desiccation for 2, 4, or 8 h in a time-dependent manner. However, desiccation for 8 h did not result in the death of all of the cells. Figure 2B,C,D show the concentrations of IL-6, IL-8, and TNF-α in the medium secreted from CEPI cells that had been stimulated by long-term desiccation. The concentrations of IL-6 and −8 gradually increased, while the concentration of TNF-α did not change. These results are in agreement with the results from the experiment on short-term desiccation.

### mRNA expression of cytokines in CEPI cells that had been subjected to long-term desiccation

**Figure 3:**
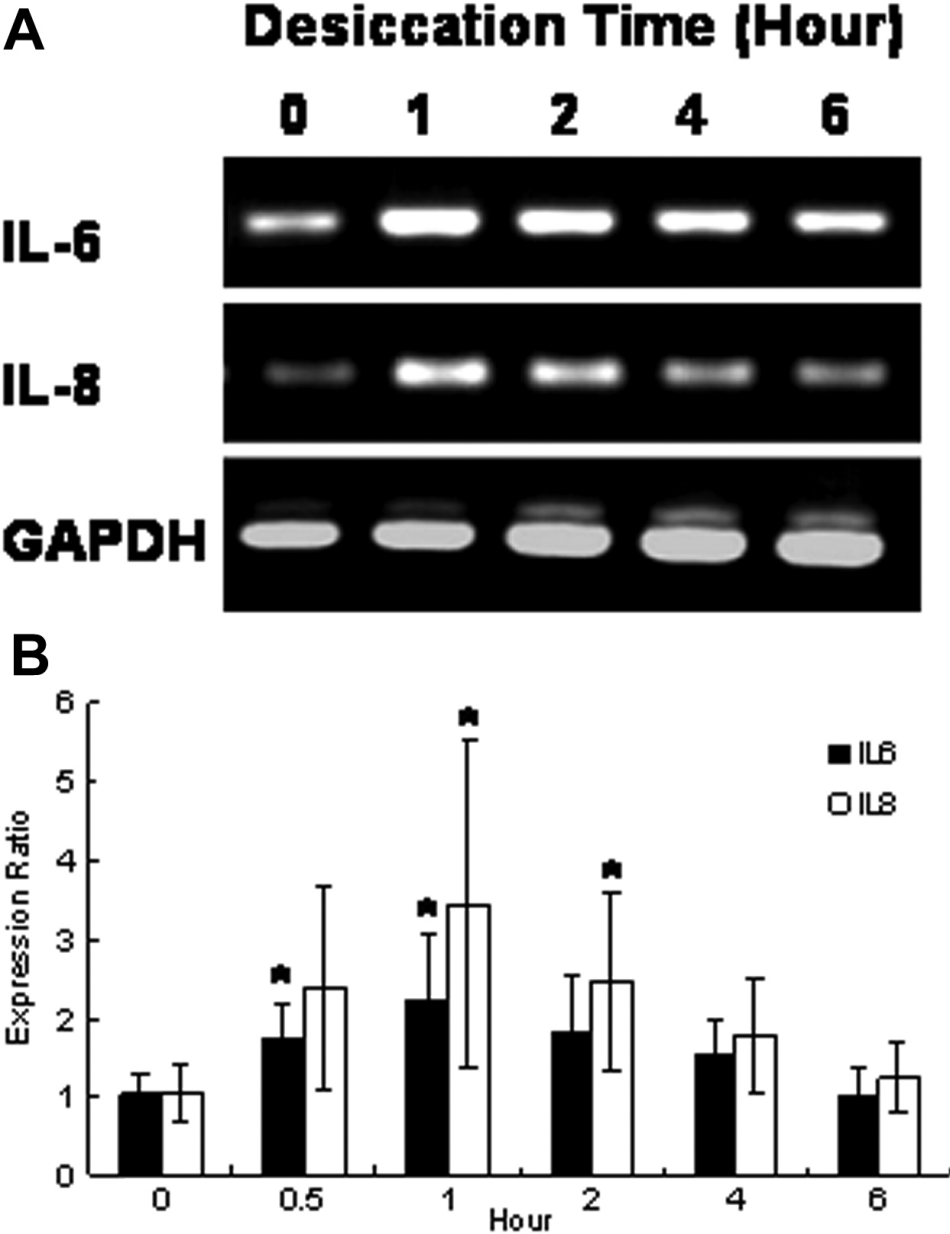
Effect of long-term desiccation on the expression of cytokines in CEPI cells. **A**: Photograph of RT–PCR results of *IL-6* and *IL-8* expression. **B**: Expression of *IL-6* and *IL-8* detected by real-time RT–PCR. The horizontal axis shows the length of time of desiccation, and the vertical axis shows the expression ratio of cytokines. The expression level at 0 h was defined as 1. Results are expressed as the mean±SD (n=6). Dunnett’s test was used to determine the significance of differences. The asterisk indicates a significant difference from the result at 0 min, p<0.05.

Because short-term and long-term desiccation increased IL-6 and/or IL-8 secretion, CEPI cells were subjected to desiccation for various lengths of time and the mRNA expressions of these cytokines in CEPI cells were estimated by RT–PCR. Expression of *IL-6* and *IL-8* increased upon 1 h desiccation; however, as the length of time of desiccation was further increased, *IL-6* and *IL-*8 mRNA expression in CEPI cells gradually declined (Figure 3A). *GAPDH* was expressed at nearly a constant level over the 6 h desiccation (Figure 3A). The mRNA levels of *IL-6* and *IL-8* were quantitatively analyzed by TaqMan real-time RT–PCR (Figure 3B). The mRNA levels of *IL-6* and *IL-8* upon 1 h desiccation were two to three times higher than the respective levels at baseline. However, the mRNA levels of *TNF-α* in CEPI cells did not change upon desiccation for up to 6 h (data not shown).

### Animal model

**Figure 4:**
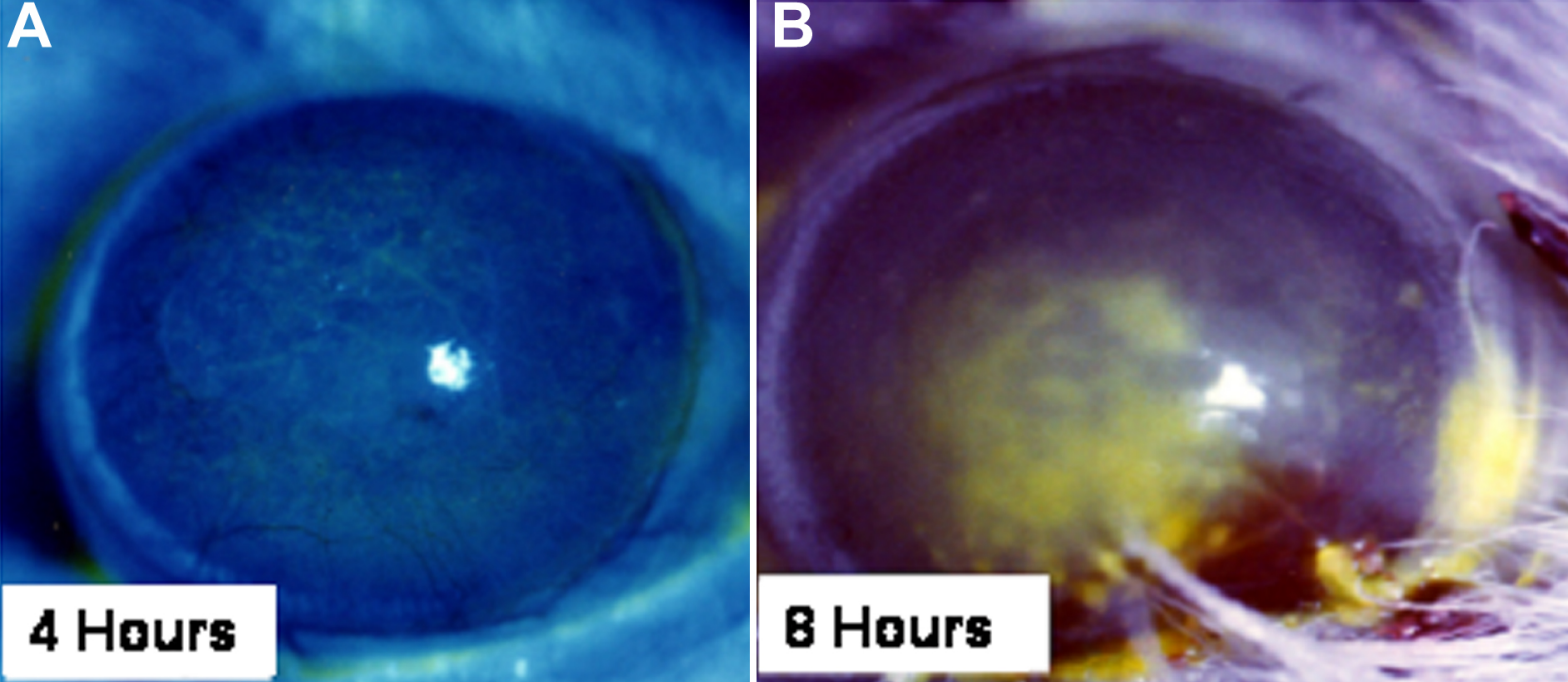
Effect of desiccation on the ocular surface of rats. **A**, **B**: Photographs of the ocular surface of rats after desiccation for (**A**) 4 or (**B**) 8 h.

To desiccate the ocular surface of rats, their eyelids were kept open by an adhesive agent after deep anesthesia. The fluorescein-stained area on the cornea increased as the desiccation time increased. Upon 8 h desiccation, the ocular surface of the rat was intensely dyed by fluorescein compared with the ocular surface upon 4 h desiccation (Figure 4A,B). After desiccation, the corneas of the dry eye model rat were collected to isolate total RNA and the levels of expression of *IL-6* and *TNF-α* were determined by TaqMan real-time PCR. Table 1 shows desiccation significantly increased the mRNA expression of *IL-6*, while it slightly increased the mRNA expression of *TNF-α*.

**Table 1:** Expression of cytokines in the rat cornea induced by desiccation.

**Gene**	**0 h**	**4 h**	**8 h**
*IL-6*	1	498±12	1472±295
*TNF-α*	1	0.83±0.17	1.16±0.34

Each value means fold change of mRNA expression, and the value at 0 h was set as 1. For example, expression of *IL-6* increased over 1,400 fold upon 8 h desiccation. Results are expressed as the mean±S.D. (n=3). The Ct of *GAPDH* was nearly constant (approximately 22) in each h.

## Discussion

Many kinds of cytokines are present in tears to maintain the condition of the ocular surface [24,25]. The proinflammatory cytokines, IL-6 and TNF-α, have also been detected in tears [32]. Pflugfelder et al. [26,27] showed that the levels of IL-6 and TNFα were increased in the conjunctival epithelium of dry eye patients. The results of the present study partially coincide with the results obtained in their study.

Specifically, we found that desiccation of CEPI cells increased the IL-6 level, similar to their results, but only slightly increased the TNF-α level (Figure 1 and Figure 2). Furthermore, in the cornea of the dry eye rat model, TNF-α slightly increased, which supported our in vitro data (Table 1). Massingale et al. [32] compared the concentration**s** of proinflammatory cytokines in tears between normal volunteers and dry eye patients, and found that IL-6 expression was more significantly increased than TNF-α expression. Similar results were obtained in the study using the scopolamine-induced dry eye model [33]. It is assumed that IL-6 is the primary proinflammatory cytokine induced by desiccation.

Dry eye stimulated the expression of TNF-α and MMP-9, and activated the MAPK signaling pathway on the ocular surface in the dry eye mouse model [34]. Conjunctival cells from dry eye patients with moderate to severe keratoconjunctivitis sicca overexpressed inflammatory and apoptosis-related markers [35]. Jozwiak et al. [36] showed that the rat corneal epithelial cell line produced inflammatory cytokines in response to proinflammatory mediators. The induction of TNF-α production may be only due to inflammation, and not due to desiccation itself. Th**e** present study also showed that desiccation increased IL-8 production in CEPI cells (Figure 2C), which was in agreement with the previous finding that the IL-8 level in conjunctiva was significantly elevated in SS patients compared with non-dry eye controls [26]. It is known that IL-8 is not existed in rats. We only analyzed the expression of IL-8 on CEPI cells which is established from human corneal epithelial cells.

Nishida et al. [37,38] hypothesized that IL-6 stimulates corneal epithelial migration by a fibronectin-dependent mechanism. If so, IL-6 induced by desiccation may also be related to the wound healing process. Whereas, Albertsmeyer et al. [39] showed that the production and release of mucins 1 and 16 were upregulated by the TNF-α and down-regulated by IL-6 in human corneal epithelial cells; this result supports that corneal injury during desiccation was partially due to IL-6.

Fabiani et al. [40] showed corneal epithelial proliferation and thickness in a dry eye model producing a controlled-environment chamber (temperature: 22.3±0.7 °C, relative humidity: 22.5±4.5%, airflow: 15 l/min). Relative humidity is low, however airflow is very slow compared with our condition, because airflow of 1m/second is approximately 1,800 l/min. It is supposed that airflow were greatly influenced in our dry eye model.

If IL-6 is one of the factors involved in desiccation-induced cell death, neutralization of IL-6 would be expected to suppress cell death. In fact, anti-IL-6 antibody protected CEPI cells from cell death induced by short-term desiccation (Figure 1E). However, application of anti-IL-6 antibody eye drops to the dry eye model rat failed to prevent corneal epithelial injury (data not shown). The amount of anti-IL-6 antibody on the ocular surface may have been insufficient, and further studies are necessary to confirm the role of IL-6 in desiccation. We conclude that desiccation induced the production of several cytokines, primarily IL-6 which causes cell death in the dry condition.

## References

[1] Craig JP, Singh I, Tomlinson A, Morgan PB, Efron N (2000). The role of tear physiology in ocular surface temperature.. Eye (Lond).

[2] Acosta MC, Tan ME, Belmonte C, Gallar J (2001). Sensations evoked by selective mechanical, chemical, and thermal stimulation of the conjunctiva and cornea.. Invest Ophthalmol Vis Sci.

[3] Korb DR, Greiner JV, Glonek T, Esbah R, Finnemore VM, Whalen AC (1996). Effect of periocular humidity on the tear film lipid layer.. Cornea.

[4] Cekiç O, Ohji M, Hayashi A, Fang XY, Kusaka S, Tano Y (2002). Effects of humidified and dry air on corneal endothelial cells during vitreal fluid-air exchange.. Am J Ophthalmol.

[5] Zuclich JA, Connolly JS (1976). Ocular damage induced by near-ultraviolet laser radiation.. Invest Ophthalmol Vis Sci.

[6] Cai CX, Birk DE, Linsenmayer TF (1998). Nuclear ferritin protects DNA from UV damage in corneal epithelial cells.. Mol Biol Cell.

[7] Shimmura S, Suematsu M, Shimoyama M, Tsubota K, Oguchi Y, Ishimura Y (1996). Subthreshold UV radiation-induced peroxide formation in cultured corneal epithelial cells: the protective effects of lactoferrin.. Exp Eye Res.

[8] Nakamura S, Matsunaga M, Saito Y, Nakashima H, Saito F, Higuchi A, Tsubota K (2003). Effect of D-beta-hydroxybutyrate on ocular surface epithelial disorder in dry eye conditions through suppression of apoptosis.. Invest Ophthalmol Vis Sci.

[9] Ubels JL, Foley KM, Rismond V (1986). Retinal secretion by the lacrimal grand.. Invest Ophthalmol Vis Sci.

[10] Ohashi Y, Motokura M, Kinoshita Y, Mano T, Watanabe H, Kinoshita S, Manabe R, Oshiden K, Yanaihara C (1989). Presence of epidermal growth factor in human tears.. Invest Ophthalmol Vis Sci.

[11] van Setten GB, Macauley S, Humphreys-Beher M, Chegini N, Schultz G (1996). Detection of transforming growth factor-alpha mRNA and protein in rat lacrimal glands and characterization of transforming growth factor-alpha in human tears.. Invest Ophthalmol Vis Sci.

[12] Yoshino K, Garg R, Monroy D, Ji Z, Pflugfelder SC (1996). Production and secretion of transforming growth factor beta (TGF-beta) by the human lacrimal gland.. Curr Eye Res.

[13] Wilson SE, Liang Q, Kim WJ (1999). Lacrimal grand HGF, KGF, and EGF mRNA levels increase after corneal epitherial wounding.. Invest Ophthalmol Vis Sci.

[14] Tsubota K, Goto E, Fujita H, Ono M, Inoue H, Saito I, Shimmura S (1999). Treatment of dry eye by autologous serum application in Sjogren’s syndrome.. Br J Ophthalmol.

[15] Mishima S, Maurice DM (1961). The oily layer of the tear film and evaporation from the corneal surface.. Exp Eye Res.

[16] Rolando M, Refojo MF, Kenyon KR (1983). Increased tear evaporation in eyes with keratoconjunctivitis sicca.. Arch Ophthalmol.

[17] Mathers WD, Binarao G, Petroll M (1993). Ocular water evaporation and the dry eye. A new measuring device.. Cornea.

[18] Craig JP, Tomlinson A (1997). Importance of the lipid layer in human tear film stability and evaporation.. Optom Vis Sci.

[19] Gendler SJ, Spicer AP (1995). Epithelial mucin genes.. Annu Rev Physiol.

[20] Argüeso P, Gipson IK (2001). Epithelial mucins of the ocular surface: structure, biosynthesis and function.. Exp Eye Res.

[21] Hicks SJ, Corfield AP, Kaswan RL, Hirsh S, Stern M, Bara J, Carrington SD (1998). Biochemical analysis of ocular surface mucin abnormalities in dry eye: the canine model.. Exp Eye Res.

[22] Davidson HJ, Kuonen VJ (2004). The tear film and ocular mucins.. Vet Ophthalmol.

[23] Nakamura Y, Sotozono C, Kinoshita S (1998). Inflammatory cytokines in normal human tears.. Curr Eye Res.

[24] Tuominen IS, Tervo TM, Teppo AM, Valle TU, Gronhagen-Riska C, Vesaluoma MH (2001). Human tear fluid PDGF-BB, TNF-alpha and TGF-beta1 vs corneal haze and regeneration of corneal epithelium and subbasal nerve plexus after PRK.. Exp Eye Res.

[25] Carreño E, Enríquez-de-Salamanca A, Tesón M, García-Vázquez C, Stern ME, Whitcup SM, Calonge M (2010). Cytokine and chemokine levels in tears from healthy subjects.. Acta Ophthalmol.

[26] Pflugfelder SC, Jones D, Ji Z, Afonso A, Monroy D (1999). Altered cytokine balance in the tear fluid and conjunctiva of patients with Sjogren's syndrome keratoconjunctivitis sicca.. Curr Eye Res.

[27] Chotikavanich S, de Paiva CS (2009). Li de Q, Chen JJ, Bian F, Farley WJ, Pflugfelder SC. Production and activity of matrix metalloproteinase-9 on the ocular surface increase in dysfunctional tear syndrome.. Invest Ophthalmol Vis Sci.

[28] Hom M, De Land P (2005). Prevalence and severity of symptomatic dry eyes in Hispanics.. Optom Vis Sci.

[29] Chia EM, Mitchell P, Rochtchina E, Lee AJ, Maroun R, Wang JJ (2003). Prevalence and associations of dry eye syndrome in an older population: the Blue Mountains Eye Study.. Clin Experiment Ophthalmol.

[30] Sharif NA, Wiernas TK, Howe WE, Griffin BW, Offord EA, Pfeifer AM (1998). Human corneal epithelial cell functional responses to inflammatory agents and their antagonists.. Invest Ophthalmol Vis Sci.

[31] Offord EA, Sharif NA, Mace K, Tromvoukis Y, Spillare EA, Avanti O, Howe WE, Pfeifer AM (1999). Immortalized human corneal epithelial cells for ocular toxicity and inflammation studies.. Invest Ophthalmol Vis Sci.

[32] Massingale ML, Li X, Vallabhajosyula M, Chen D, Wei Y, Asbell PA (2009). Analysis of inflammatory cytokines in the tears of dry eye patients.. Cornea.

[33] Viau S, Maire MA, Pasquis B, Grégoire S, Fourgeux C, Acar N, Bretillon L, Creuzot-Garcher CP, Joffre C (2008). Time course of ocular surface and lacrimal gland changes in a new scopolamine-induced dry eye model.. Graefes Arch Clin Exp Ophthalmol.

[34] Luo L, Li DQ, Doshi A, Farley W, Corrales RM, Pflugfelder SC (2004). Experimental dry eye stimulates production of inflammatory cytokines and MMP-9 and activates MAPK signaling pathways on the ocular surface.. Invest Ophthalmol Vis Sci.

[35] Brignole F, Pisella PJ, Goldschild M, De Saint Jean M, Goguel A, Baudouin C (2000). Flow cytometric analysis of inflammatory markers in conjunctival epithelial cells of patients with dry eyes.. Invest Ophthalmol Vis Sci.

[36] Józwiak J, Skopinski P, Malejczyk J (2000). Production of interleukin-1 beta, interleukin-6 and tumor necrosis factor-alpha by a rat corneal epithelial cell line.. Int J Tissue React.

[37] Nishida T, Nakamura M, Mishima H, Otori T (1992). Interleukin 6 promotes epithelial migration by a fibronectin-dependent mechanism.. J Cell Physiol.

[38] Nakamura M, Nishida T (1999). Differential effects of epidermal growth factor and interleukin 6 on corneal epithelial cells and vascular endothelial cells.. Cornea.

[39] Albertsmeyer AC, Kakkassery V, Spurr-Michaud S, Beeks O, Gipson IK (2010). Effect of pro-inflammatory mediators on membrane-associated mucins expressed by human ocular surface epithelial cells.. Exp Eye Res.

[40] Fabiani C, Barabino S, Rashid S, Dana MR (2009). Corneal epithelial proliferation and thickness in a mouse model of dry eye.. Exp Eye Res.

